# Ability of a dynamical climate sensitive disease model to reproduce historical Rift Valley Fever outbreaks over Africa

**DOI:** 10.1038/s41598-024-53774-x

**Published:** 2024-02-16

**Authors:** Alizée Chemison, Gilles Ramstein, Anne Jones, Andy Morse, Cyril Caminade

**Affiliations:** 1 Laboratoire des Sciences du Climat et de l’Environnement (LSCE), CEA, CNRS, UVSQ, 91190 Gif-sur-Yvette, France; 2 IBM Research Laboratory, Daresbury, WA4 4AD UK; 3 Department of Geography and Planning, School of Environmental Sciences, University of Liverpool, Liverpool, L69 7ZT UK; 4 Earth System Physics, Abdus Salam International Centre for Theoretical Physics, 34151 Trieste, Italy

**Keywords:** Viral infection, Projection and prediction

## Abstract

Rift Valley Fever (RVF) is a zoonosis transmitted by *Aedes* and *Culex* mosquitoes, and is considered a priority pathogen by the WHO. RVF epidemics mostly occur in Africa and can decimate livestock herds, causing significant economic losses and posing health risks for humans. RVF transmission is associated with the occurrence of El Niño events that cause floods in eastern Africa and favour the emergence of mosquitoes in wetlands. Different risk models have been developed to forecast RVF transmission risk but very few studies have validated models at pan-African scale. This study aims to validate the skill of the Liverpool Rift Valley Fever model (LRVF) in reproducing RVF epidemics over Africa and to explore the relationship between simulated climatic suitability for RVF transmission and large-scale climate modes of variability such as the El Niño Southern Oscillation (ENSO) and the Dipole Mode Index (DMI). Our results show that the LRVF model correctly simulates RVF transmission hotspots and reproduces large epidemics that affected African countries. LRVF was able to correctly reproduce major RVF epidemics in Somalia, Kenya, Zambia and to a lesser extent for Mauritania and Senegal. The positive phases of ENSO and DMI are associated with an increased risk of RVF over the Horn of Africa, with important time lags. Following research activities should focus on the development of predictive modelling systems at different time scales.

## Introduction

Rift Valley fever (RVF) is a zoonotic mosquito-borne disease that was first isolated and discovered in Kenya in 1931^1^. Rift Valley fever virus (RVFV) belongs to the genus *Phlebovirus* in the family *Bunyaviridae*^2^. Rift Valley Fever is a vector-borne disease that primarily affects goats and sheep but can also infect cattle, camels, other wildlife animals (antelopes and wildebeest) and humans^3^. Epizootics of RVF are characterized by abortion storms, increased mortality rates in livestock and significant economic losses. Infection experiments showed that the mortality rate in new-born lambs can reach 95-100% while it usually ranges between 20 and 30% in adult sheep^4^. Abortion rate in pregnant ewes can be as high as 100%. Recovered animals usually maintain lifelong immunity^5^. RVF virus is primarily transmitted to animals by the bite of infected *Aedes* or *Culex* mosquitoes^6^. Humans can also be infected by mosquito bites and by direct contact with infected animal materials (viraemic blood, infected organs, foetuses, consumption of raw meat and to a lesser extent unpasteurized milk). Slaughterhouse and abattoir personnel, herders and veterinarians usually face a greater risk of infection. The human symptoms are characterized by high fever, headaches, dizziness, back pain and liver abnormalities. In some cases (8–10%), a severe and often a fatal haemorrhagic form of the disease can occur with ocular complications, meningoencephalitis and haemorrhagic fever^7^. Currently, there are commercially available vaccines (mostly live-attenuated and inactivated) for animals and an unlicensed human vaccine has been clinically tested on most at-risk professionals such as veterinarians, livestock farmers and slaughterhouse personnel^8^.

One of the first well-documented RVF epidemic occurred in western provinces of South Africa in autumn 1951 and affected sheep, cattle, probably wild bucks, and humans^9^. In 1977–1978, a large RVF epidemic caused about 600 human deaths in Egypt. Imported livestock from the Horn of Africa might have triggered this epidemic and increased abundance of mosquito vectors from the flooded Nile might have played an amplification role^10^. The 1997–1998 RVF epidemic that caused thousands of livestock deaths and about 500 human unexplained deaths in northeastern Kenya and southern Somalia has been associated with the occurrence of El Niño-and local floods^11,12^. For the first time, about 880 RVF human cases and 124 deaths were reported outside the African continent, in Saudi Arabia in 2000–2001^13^. Importation of infected livestock is believed to have triggered the outbreak^14^. In 2006-2007, a large RVF epidemic affected Sudan, Somalia, Kenya and Tanzania causing substantial deaths in livestock and about 900 human casualties^15–18^. Over southern Mauritania and northern Senegal, there is a large number of domestic animals and the presence of temporary ponds modulated by the summer rainy season. In Mauritania, the first documented RVF outbreak occurred in 1987 with about 220 reported human deaths^19^. Several other outbreaks followed in 1993^20^, 1998^21^, 2003^22^, 2010^23^, 2012^24^, 2013–2014^25^ and 2015^26^ across Senegal and Mauritania. The first reported RVF outbreak in Niger occurred in 2016, with 266 suspected human cases, 33 human deaths and significant loss of livestock^27^.

Consequently, RVF became a significant public health issue. According to the American Centers for Disease Control (CDC) and the World Organisation for Animal Health (WOAH), RVF virus is a high impact and top priority pathogen with high potential for setback in livestock trade^28^. In 2018, the WHO also listed RVFV as a priority pathogen, with significant epidemic or pandemic potential in future, alongside COVID-19, Crimean-Congo haemorrhagic fever, Ebola and Marburg virus diseases, Lassa fever, Middle East respiratory syndrome (MERS) and Severe Acute Respiratory Syndrome (SARS), Nipah and henipaviral diseases, Zika virus and Disease X^29^.

The largest RVF epidemics in eastern Africa have been associated with floods caused by the warm phase (El Niño) of the El Niño Southern Oscillation (ENSO)^30^. Floods create suitable conditions for competent RVF mosquitoes over large African plains (dambos). *Aedes* and *Culex* genera are considered to be the main vectors of RVF in Africa^31^. *Aedes* are the primary vectors of RVF, their abundance usually peaks during the rainy season, and they can transmit the virus transovarially to their offspring^6^. Hence, *Aedes* mosquitoes are usually assumed to be reservoirs of RVFV in Africa. *Culex* mosquitoes must bite an infected host to become infectious. They usually lay eggs on the surface of water bodies, and their abundance increases during periods of heavy rainfall^30^ and can therefore amplify RVF transmission locally. In Senegal and Mauritania, the impact of ENSO on RVF epidemics is not evident. However, the occurrence of floods at the end of the standard rainy season (Oct–Nov) has been associated with increased RVF transmission risk^32,33^.

Mathematical models are useful tools to investigate the impact of environmental and socio-economic factors on disease transmission risk. Two main modelling approaches are usually employed. On one hand, Ecological niche models (or phenomenological model or statistical models) allow to develop our understanding of complex systems, to derive patterns and information from data, without necessarily focusing on the underlying mechanisms at play^34^. Statistical models are derived directly from observations and utilize fitting methods that have their own uncertainty. They cannot extrapolate risk beyond those within the training dataset. These models have however been successfully used to map niche of species, pathogens and diseases^35^. On the other hand, mechanistic (or dynamical) models can be utilized when observed data is missing and they have been employed to forecast disease risk at different time scales^36^. These models require detailed knowledge about vector and pathogen processes to parameterize the model. However, relationships between environmental factors and disease risk are often derived from a collection of small-scale studies with large associated uncertainties. Importantly, a dynamical empirical risk model based on satellite-based vegetation, Sea Surface Temperature, Outgoing Long Wave Radiation (OLR) and rainfall data successfully predicted the RVF epidemic that affected Tanzania in 2007^37^. Some 49 different mechanistic RVF models have recently been discussed in an extensive literature review exercise^36^. Most RVF models mainly aimed at exploring epidemiological mechanisms, at evaluating efficiency of control strategies, or at exploring the consequences of hypothetical epidemics on animal and human populations. Only one study^38^, based on the Liverpool Rift Valley Fever (LRVF) model^39^, examined the impact of climate change scenarios on future RVF transmission risk over eastern Africa. Importantly, this model was parameterized and validated using surveillance data for Kenya before risk was extrapolated to the whole East African region.

The Food and Agriculture Organization of the United Nations routinely use RVF models to provide forecasts and timely alerts on the risk of Rift Valley Fever outbreaks^40^. However, very few studies have validated mathematical RVF risk models at pan-African scale over long periods due to the paucity of observed data. In addition, studies show that El Niño and DMI events have large-scale climate impacts^41^ but the relationship between ENSO, the DMI and RVF outbreaks in Africa has primarily been studied at country and local scales. Hence, the objectives of this study are two-fold. First, we use observed data available from WOAH and from another published review^42^ to determine the forecasting skill of the LRVF model in reproducing historical RVF epidemics at country scale for Africa over a 38 year time period. Second, we explore the theoretical relationship between simulated disease prevalence and large-scale climatic modes of variability, such as ENSO and DMI, and their impact on regional climatic conditions. Finally, we provide recommendations and future perspectives of this work.

## Results

### Validation of LRVF simulations

**Figure 1:**
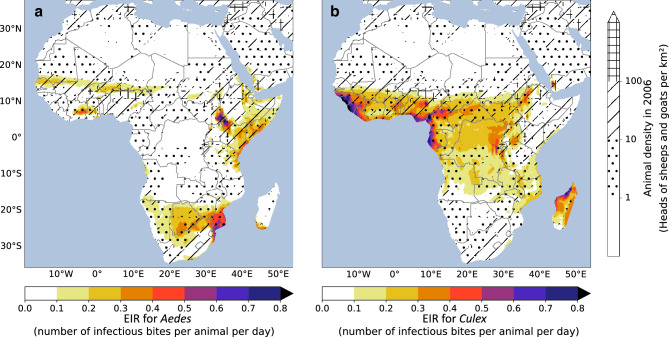
Simulated EIR for (**a**) *Aedes* and (**b**) *Culex* in number of infectious bites per animal per day, averaged over the period 1979–2017 (color shading) superimposed on the number of goats and sheep per km^2^ for the year 2006. The dots correspond to animal density ranging between 1 and 10 animals per km^2^, the crosshatch to a density ranging between 10 and 100 animals per km^2^ and the grid to a density above 100 animals per km^2^. Figure generated with python 3.8.6 (https://www.python.org/downloads/release/python-386/).

Figure 1 shows the annual means of simulated EIR for *Aedes* (left) and *Culex* (right) for the period 1979–2017. Density of small ruminant hosts (sheep and goats) are superimposed on simulated EIRs. On average, simulated EIRs values for *Aedes* are large over southern Africa (Botswana, Angola, Zimbabwe, Mozambique, and southern Madagascar), the Horn of Africa (eastern Kenya, north-eastern and coastal Tanzania, Somalia, southern Sudan, Uganda and Rwanda) and over the northern fringe of the Sahel over West Africa (northern Senegal, southern Mauritania, central Mali and southern Niger). Overall, the intersect between simulated EIR hotspots for *Aedes* and large livestock densities match observed circulation of RVFV. RVF epidemics have been reported over northern Senegal and southern Mauritania, where pastoralist communities and large animal densities are present^36^. RVFV circulation in livestock animals has also been reported over central and southern Mali^43^. Hotspots over Zimbabwe, and the eastern part of the Horn of Africa also match observed circulation of RVFV^42^.

Importantly, simulated EIRs for *Aedes* by the LRVF model are consistent with other model estimates based on satellite imagery^30,44^. Simulated EIRs for *Culex* match annual input rainfall distribution pattern over Africa (spatial correlation = 0.55, significant value at the 99.5% CI) (Fig. 1, right). Large EIR values match rainfall maximum over the western part of Africa, coastal areas surrounding the Gulf of Guinea (Cameroon, Gabon, southern Nigeria), central Africa and Madagascar (Fig. S1). EIR hotspots for *Culex* are consistent with LRVF model parameterization as *Culex* mosquitoes lay their eggs at the surface of large water bodies.

The average seasonal cycle of simulated prevalence in immature animals (Fig. S2) and EIRs for *Culex* mosquitoes (Fig. S3) lags rainfall seasonality by about 2–3 months (Fig. S1). Maximum values are simulated over the northernmost part of the Sahel during Sep–Oct–Nov, where the typical rainfall season usually ranges from July to September. Large values are also simulated during Mar–Apr–May south of the equator, where largest rainfall usually occurs between December and February (Fig. S2–S3). The mean seasonal cycle of simulated EIR for *Aedes* is in phase with the rainy season (Fig. S4). This finding is consistent with the fact that *Aedes* mosquitos’ abundance tend to peak during the rainy seasons in Africa. Largest EIR values are simulated over fringe regions. Over West Africa, the northernmost part of the Sahel show the largest EIR values during and following the rainy season (Jun–July–Aug and Sep–Oct–Nov). Consistently, the largest RVF epidemics have been reported during the second half and late part of the rainy season (Oct-Nov) in Senegal and Mauritania^32^. Largest EIR values are simulated during the short (Oct–Nov–Dec) and long (Mar–Apr–May) rainy seasons over eastern Africa. Over southern Africa, the highest EIR values for *Aedes* are shown for the austral rainy season from December to February.

**Figure 2:**
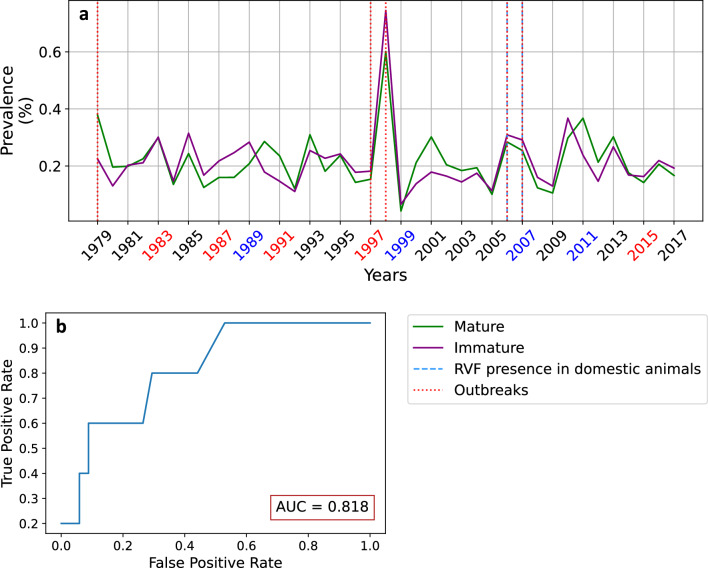
(**a**) Simulated prevalence in mature (green solid line) and immature (purple solid line) livestock (based on ERA5 driving climate data) superimposed on observed outbreaks in Kenya from Nanyingi et al.^42^ (red dotted line) for the period 1979–2014. Presence of the virus detected in domestic animals is depicted by the blue dashed line for the period 2005–2018; see methods for more information. (**b**) Associated ROC curve and AUC score. Figure generated with python 3.8.6 (https://www.python.org/downloads/release/python-386/).

Overall, the LRVF model tend to reproduce observed RVF hotspots over Africa. In a second step we evaluate the capability of the LRVF model in reproducing historical RVFV circulation at country scale using data available from the published literature^42^ and WOAH. Time series of simulated prevalence in mature and immature livestock for Kenya (Fig. 2) and other African countries (Fig. S5) are compared to the occurrence of observed outbreaks and circulation of RVFV in wild and domestic animals. The reported RVF epidemics in 1979, 1998 and 2006–2007 over Kenya are well reproduced by the LRVF model (Fig. 2). Notably, the largest RVF epidemic occurred during the El Niño event of 1997–1998 when the largest peak in prevalence is simulated (Fig. S5). However, the model predicted large RVF prevalence values in Kenya in 2010–2011, when no circulation of RVFV was reported (Fig. 2). The associated AUC score is about 0.82 for Kenya, denoting good forecasting skill of the LRVF model driven by ERA5 reanalysis for the period 1979–2017. The LRVF model was able to reproduce 3 hits, 31 correct rejections, 3 false alarms and 2 misses over Kenya for a 39y period (Table S1). This finding is not surprising because the LRVF model was originally parameterized and calibrated using observed surveillance data for Kenya and Tanzania (see methods). Notably, good AUC scores are also shown for Somalia (0.90), Zambia (0.69) and to a lesser extent over Senegal (0.61) and Mauritania (0.65) (Table 1). Over Tanzania, AUC value is lower (0.55), but the model also simulates the largest peak in prevalence in immatures during El Niño 1997–1998 (Fig. S5). A false alarm would have been issued by the model in 2001, and spikes in prevalence are also shown in 2006–2007 when a large RVF e pidemic affected Tanzania^37^. The best AUC is shown for Somalia, with one of the largest prevalence peak occurring again in 1997–1998. Over Senegal and Mauritania, the LRVF model reproduce some of the years affected by RVF, but also simulates several false alarms (see Table S1). Over Egypt, Zimbabwe and Sudan, AUC values are below 0.5, implying that the model is not better than a random guess.

Similar analysis were carried out using the EWEMBI climate dataset as input to the LRVF model and are presented in supplementary materials in Figs. S6–S7–S8–S9–S10–S11. Precipitation patterns slightly differ between both datasets (Figs. S1–S7). Overall, simulated prevalence and EIR values are larger in EWEMBI (Figs. S6–S8–S9–S10) with respect to ERA5 simulations (Figs. 1, S2–S3–S4). In terms of AUC, the results are close and show that the LRVF model captures several historical epidemics over Somalia, Kenya, Mauritania, Zambia and Senegal (Table S1). Overall, AUC scores are larger in EWEMBI simulations with respect to ERA5 simulations (Table 1). However, the model driven by ERA5 data has better AUC scores over Kenya and Zambia (Table 1).

**Table 1:** AUC and optimal threshold for immature livestock in each country and two driving climate datasets.

Country	AUC ERA5	Prevalence threshold ERA5	AUC EWEMBI	Prevalence threshold EWEMBI
Senegal	0.615	3.35E−01	0.660	4.05E−01
Mauritania	0.652	1.46E−03	0.720	2.08E−02
Egypt	0.421	1.40E−04	0.230	2.15E−04
Kenya	0.818	2.85E−01	0.727	3.84E−01
Zimbabwe	0.361	4.40E−01	0.364	9.72E−02
South Africa	0.538	1.27E−01	0.740	9.58E−02
Madagascar	0.569	9.74E−01	0.594	1.16E+00
Sudan	0.489	4.77E−02	0.632	2.01E−01
Somalia	0.900	1.44E−01	0.963	1.05E−01
Zambia	0.697	3.62E−01	0.635	4.86E−01
Tanzania	0.550	9.77E−01	0.773	7.46E−01

### Relationship between simulated RVF prevalence, ENSO and DMI

**Figure 3:**
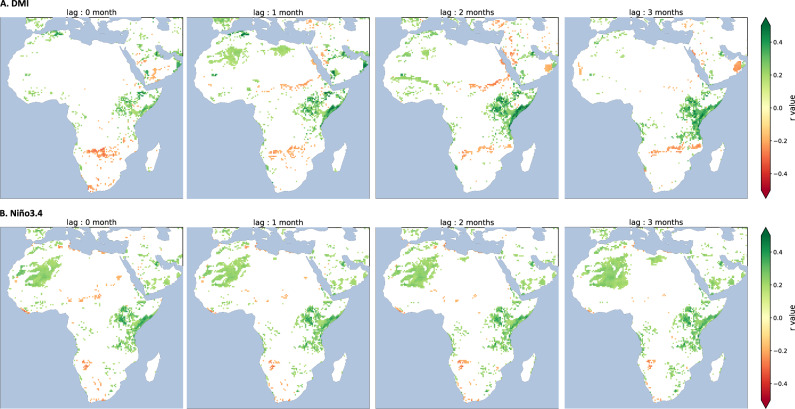
Lagged correlations (r) between the DMI index (top), the Niño3.4 index (bottom) and simulated prevalence in immature livestock during the Dec–Jan–Feb season for the period 1979–2017. The SST indices have no lags (left panels), then the SST indices are leading simulated prevalence by 1 month (left middle panels), 2 months (right middle panels) and 3 months (right panels). Only correlations significant at the 95% CI are shown. Figure generated with python 3.8.6 (https://www.python.org/downloads/release/python-386/).

Figure 3 depicts the lagged correlation coefficients between simulated prevalence in immature livestock and Niño3.4 and DMI indices for different time lags. SST indices are leading simulated prevalence because there is an inherent biological lag between the occurrence of El Niño events, associated floods and subsequent reporting of RVFV circulation in a given region. On average during the Dec–Jan–Feb season (i.e. boreal winter), largest prevalence values in immature livestock are simulated over the eastern coasts of the Horn of Africa, eastern Madagascar, central Africa and the coasts of Gabon and Cameroon (Fig. S2). Large positive correlations are shown between the Niño3.4 index and simulated prevalence over the coasts of Eastern Africa (Fig. 3) at lag 1mo to lag 3mo. Similar patterns are shown for correlations between the DMI index and simulated prevalence (Fig. 3). Hence, positive phases of ENSO (El Niño events) and DMI are associated with an increased risk of RVF over the Horn of Africa, and vice-versa. The lags denote that ENSO events developing during boreal autumn already have an impact on simulated prevalence during the following boreal winter season.

In Mar–Apr–May (i.e. boreal spring), largest prevalence values in immature livestock are simulated over southern-central, austral Africa and Madagascar (Fig. S2). Large positive correlations between Niño3.4 and simulated prevalence are shown over the southern Rift region (southern Kenya, Tanzania, coasts of Mozambique), southern DRC and Angola in Mar–Apr–May (Fig. S12). These correlation values are largest when the SST indices are leading simulated prevalence by 4–6 months, denoting the influence of large-scale SST events occurring during the previous boreal winter. In Sep–Oct–Nov, simulated prevalence values are largest over the Sahel (Fig. S2). Negative correlations are depicted between the Niño3.4 index and simulated prevalence over the Sahel (Fig. S13) at lag 1mo to lag 3mo. El Niño events can develop during late boreal spring and summer, but they tend to peak the following boreal winter. El Niño tends to be associated with drier than average conditions over the western part of the Sahel, but this relationship is not stationary^45^ and not as robust as the relationship between El Niño and induced floods over eastern Africa.

Prevalence anomalies between El Niño and La Niña years are consistent with these findings (Fig. S14). Simulated prevalence in immature livestock tends to increase over the eastern coasts of the Horn of Africa, Zimbabwe, Mozambique and Botswana, while it tends to decrease over the western Sahelian fringe, parts of Ethiopia and South Africa during El Niño events. Correlation maps between SST indices and rainfall data are presented in Supplementary Materials (Figs. S15–S16–S17). Similar linear relationships are highlighted, namely positive correlations between positive phases of ENSO, DMI and rainfall over eastern Africa contrasting with negative correlations over the Sahel. These results underline the importance of rainfall variability in driving simulated RVF hotspots in LRVF.

## Discussion

The LRVF model only driven by observed climate variables was able to reproduce key historical RVF epidemics over Kenya, Somalia and Zambia. The AUC scores were moderate for Senegal and Mauritania. For other African countries, the skill of the LRVF model was low. Large prevalence values were simulated over the Rift region of Africa during the large El Niño 1997–1998 event that had large impacts on livestock and population welfare. This well-known relationship is consistent with former published studies^37^. Interestingly, large positive correlations were also depicted between the Indian Dipole Mode and simulated prevalence over the Horn of Africa with time lags. This finding is consistent with the demonstrated impact of the positive phase of the Indian Ocean Dipole on extreme rainfall over eastern Africa during the short rain season^46^. On average, the overlap between simulated EIR for *Aedes* and livestock density provides realistic RVF hotspots over the African continent.

It is noteworthy that the LRVF model was only driven by climate variables and several other important factors are not considered in the modelling framework. Dynamic animal movements were not considered and are critical for disease (re)emergence in new regions^36^. For example, the epidemics in Saudi Arabia and in Egypt were caused by the importation of infected animals^13,47,48^. Cattle movements between Comoros and Madagascar very likely caused the (re)emergence of RVF in Madagascar, environmental factors might have modulated RVFV transmission locally^49^. Vulnerability and other socio-economic factors were not factored in, and they can also have large impacts on RVF risk of RVF emergence locally.

Given the potential skill of the LRVF model in reproducing past epidemics and RVFV circulation in some African countries, following research activities should focus on developing forecasting modelling systems. First, the LRVF model could be driven by ensemble seasonal forecast models to produce risk bulletins for the upcoming risk season (2–3 months in advance). For example, the Liverpool Malaria Model driven by seasonal climate forecasts (from the European Centre for Medium-Range Weather Forecasts’ System 4) was able to reproduce 6/7 upper tercile malaria seasons in Botswana over the 1982–2006 period^50^. Second, standard climate (Representative Concentration Pathways) and population (Shared Socio-economic Pathways) scenarios could be employed to forecast RVF risk over longer time scales. Climate change scenarios simulate a large increase in future rainfall over eastern Africa^51^ and such changes could potentially impact RVF epidemics in future. LRVF was used to simulate future RVF transmission risk based on two climate change scenarios with a single General Circulation Model (GCM)^38^. This study highlighted that RVF outbreaks could potentially spread further in parts of eastern Africa to date unaffected by the disease. However, the LRVF model could be driven by a larger ensemble of GCMs and novel RCP-SSP scenarios produced for the 6th assessment report of the Intergovernmental Panel on Climate Change. Risk over temperate regions in a warmer climate could also be investigated. For example, some studies estimate that RVFV could spread into Europe^52^. The impact of climate tipping points, such as a rapid ice-sheet destabilization of the Arctic could cause a large reorganisation of the ocean dynamics by the Atlantic meridional overturning circulation with significant impacts on tropical hydrology^53^ and could drastically modify RVF distribution. Rapid melting of the Arctic could lead to a southward shift of the rain-belt in Africa, with potential large impacts on agriculture^54^ and vector-borne disease risk^55^. Finally, the use of fully susceptible sentinel animals and the improvement of surveillance systems in Africa will be key in preventing future epidemics and spread of RVFV. This data can be used in conjunction with predictive risk models to inform vector control and surveillance activities. Such multi-disciplinary work should be conducted using a One Health approach, and large multi disease model comparison exercise should also be encouraged^56^.

## Methods

### Liverpool rift valley fever model

The Liverpool Rift valley Fever model (LRVF) is used to simulate RVF transmission risk. LRVF was mathematically and structurally adapted from the Liverpool Malaria Model^57^. The LRVF considers two distinct generic genera of mosquitoes, which have different life cycles: *Aedes*, the primary vector and reservoir of the disease, and *Culex*, the secondary vector which is responsible for epidemic amplification. Livestock hosts are divided into two age categories: young and adult. The resistance of adults to this virus is generally much higher than young animals; hence, this underlines the importance of separating mature and immature host stages^38^.

LRVF follows a deterministic SEIR compartmental approach to the epidemiology of RVF. Vectors and hosts are categorised according to their epidemiological status: ’susceptible (S)’, ’exposed (E)’ and ’infectious (I)’. In livestock, an additional category, “recovered and immune (R)”, is included for hosts that have been infected and survived because they have developed lifelong immunity^5^. The life cycle of *Culex* mosquitoes includes three categories: eggs, immatures (larvae-pupae) and “susceptible” adults. Ovoposition depends on temperature and rainfall; mosquito survival only depends on temperature. For the life cycle of *Aedes* vectors, eggs are deposited on dry or humid substrates, precipitation is necessary to reach egg maturation before adults subsequently emerge. The gonotrophic cycle depends on temperatures. Mortality of eggs, larvae and adults also depends on temperature. Vertical transmission of RVFV is only modelled in infectious *Aedes* females. Contacts between livestock hosts and vectors also rely on temperature^39^.

The vector module of LRVF was parameterised using Entomological Inoculation Rate (EIR) data collected in both Garissa district, Kenya and Arusha in Tanzania in 1997/98 and 2006/07^38,39,58^. Observed EIR data was derived from field studies carried out in the Ijara district of Kenya within the HEALTHY FUTURES European project framework^38,59^.

Input parameters for LRVF are daily gridded precipitation and temperature data. Model outputs are available on the same spatio-temporal grid than the input data. As the LRVF model was only driven by observed daily rainfall and temperature, it somehow simulates climatic suitability for RVF transmission risk, other important socio-economic factors are not accounted for. Output variables include prevalence in immature and mature hosts, EIR for both *Culex* and *Aedes* mosquitoes, and abundance estimates of vectors at different life stages. The LRVF model solely depends on climatic conditions and simulated livestock immunity. The consideration of natural immunity is paramount for RVF, hence it is necessary to consider past climatic and transmission events as well as the turnover time of livestock^39^. Parameterization of the livestock host module was derived using Ijara District community-based participatory survey that was conducted by scientists at the International Livestock research Institute in Kenya^59^. In this study, we mostly focus on simulated prevalence in mature and immature livestock, as well as the simulated EIR of *Aedes* and *Culex* mosquitoes, which corresponds to the number of infectious bites per day per host.

### Data

#### Input climate data

LRVF was driven by total daily precipitation and temperatures at 2 metres, from the ERA5 reanalyses. ERA5 data is available at daily time step with a spatial resolution of 0.5^∘^ x 0.5^∘^ for the period 1979-2017^60^.

In order to further improve the validation of the model, the same analyses were performed using the EWEMBI dataset with daily precipitation and temperature data also on a 0.5^∘^ x 0.5^∘^ grid over the period 1979-2016^61^.

#### Animal density data

The animal density data was obtained from livestock.geo-wiki.org^62^. This dataset, available on a 5km x 5km grid, represents the number of animals per km^2^ for 2006^63^. Goat and sheep densities are shown separately in supplementary materials (Fig. S18) and were summed in Figure 1.

#### Observed epidemic and serology data

To validate LRVF simulations, annual country-scale binary epidemic data, was derived from Nanyingi et al.^42^ for the period 1979–2014. We complemented this dataset, with wild and domestic animal serology data from WOAH for the period 2005–2017^64^.This data is publicly available from the World Animal Health Information System (WAHIS) platform by WAHO [available at https://wahis.woah.org/#/dashboards/country-or-disease-dashboard]. This dataset provides presence/absence of RVF in wildlife or domestic animals; at a six monthly time step from 2005 until 2017 (categories include “presence”, “absence”, “suspected” and “no information” status at country scale). We have included data extracted for “World Region = Africa” and “Disease = Rift Valley Fever virus”. We only considered positives when disease status was reported as “present”. The data is based on official reports (immediate notifications and follow-up reports, six-monthly reports and annual reports) submitted by the relevant Veterinary Services through WAHIS. Simulated immature prevalence data by the LRVF was then compared to the aforementioned observed estimates. We calculated true positive rates (TPR) and false positive rates (FPR) by varying the prevalence thresholds in immature animals for which the model considers the presence of an epidemic. Then, we calculated the Receiver Operating Characteristic (ROC) curve and calculated the associated area under the curve (AUC) (Table 1). An AUC close to 0.5 depicts a random model while an AUC close to 1 depicts a perfect predictive model^65^.

#### ENSO and DMI indices

The monthly Sea Surface Temperature (SST) indices used in this study are Niño3.4^66^, and the Dipole Mode Index (DMI)^67,68^. The Niño3.4 represents phases of the El Niño Southern Oscillation (ENSO) in the Pacific Ocean. Its calculation is based on the SST anomaly averaged over the area from 5S–5N and 170–120W^66^. The DMI index represents the Indian Dipole Mode across the southern Indian Ocean^67^. It is represented by anomalous SST gradient between the western equatorial Indian Ocean (50E–70E and 10S–10N) and the southeastern equatorial Indian Ocean (90E–110E and 10S–0N). Monthly SST anomalies with respect to the 1981-2010 period were used. These indices were obtained from the National Oceanic and Atmospheric Administration (NOAA) at (https://psl.noaa.gov/gcos_wgsp/Timeseries/). The Niño3.4 index is available from the Physical Science Laboratory (PSL) and based on the HadISST1 dataset^66^. The DMI index is available from NOAA/PSL and based on the HadISST1.1 dataset^68^. Simulated prevalence and EIRs were correlated with these SST indices to determine the relationship between large-scale climatic oscillations and simulated RVF epidemics over Africa. Only significant correlations at the 95% confidence interval (Student t-test) are depicted in the following. We also used a composite analysis to determine anomalies for El Niño and La Niña years. For El Niño, strong events, corresponding to the years 1987–1988, 1991–1992, and very strong events, 1982–1983, 1997–1998, 2015–2016, were retained. For La Niña, only years corresponding to strong events were selected: 1988–1989, 1998–1999, 1991-2000, 2007–2008, 2010–2011. This selection was based on NOAA/PSL criterion^69^.

### Supplementary Information

Supplementary Information.

## Data Availability

All supporting simulations are available in the public repository “Data files to reproduce the paper: Ability of a dynamical climate sensitive disease model to reproduce historical Rift Valley Fever outbreaks over Africa” available at https://osf.io/vyqg4/?view_only=59faa0792d294af1a351c46471843e1d.
