# Supplementary material for: Ability of a dynamical climate sensitive disease model to reproduce historical Rift Valley Fever outbreaks over Africa
Source: Sci Rep. 2024 Feb 16;14:3904. doi: 10.1038/s41598-024-53774-x (PMC10873308; doi:10.1038/s41598-024-53774-x)
Supplement: Supplementary file 1 — Supplementary Information. [file 41598_2024_53774_MOESM1_ESM.pdf]

Ability of a dynamical climate sensitive disease model to reproduce historical Rift Valley Fever outbreaks over Africa : Supplementary materials

Alizée Chemison[1,+], Gilles Ramstein[1], Anne Jones[2], Andy Morse[3], and Cyril Caminade[4,*,+]

[1]Laboratoire des Sciences du Climat et de l'Environnement (LSCE), CEA, CNRS, UVSQ, Gif-sur-Yvette, 91190, France
[2]IBM Research Laboratory, Daresbury, UK, WA4 4AD
[3]Department of Geography and planning, School of Environmental Sciences, University of Liverpool, Liverpool, UK, L69 7ZT
[4]Earth System Physics, Abdus Salam International Centre for Theoretical Physics, Trieste, Italy, 34151
[*]ccaminad@ictp.it
[+]these authors contributed equally to this work

	ERA5				EWEMBI			
**Country**	**Hit**	**Correct rejection**	**False alarm**	**Miss**	**Hit**	**Correct rejection**	**False alarm**	**Miss**
Senegal	7	21	7	4	8	17	10	3
Mauritania	15	8	15	1	11	15	8	4
Egypt	1	16	22	0	1	8	29	0
Kenya	3	31	3	2	3	30	3	2
Zimbabwe	1	30	5	3	3	13	21	1
South Africa	2	30	2	5	6	23	8	1
Madagascar	3	28	4	4	3	25	6	4
Sudan	4	11	24	0	2	29	5	2
Somalia	3	33	2	1	4	30	4	0
Zambia	1	26	12	0	1	23	14	0
Tanzania	1	34	0	4	4	27	6	1

Table S1. Contingency table, per country for ERA5 (1979-2016) and EWEMBI (1979-2017) LRVF simulations.

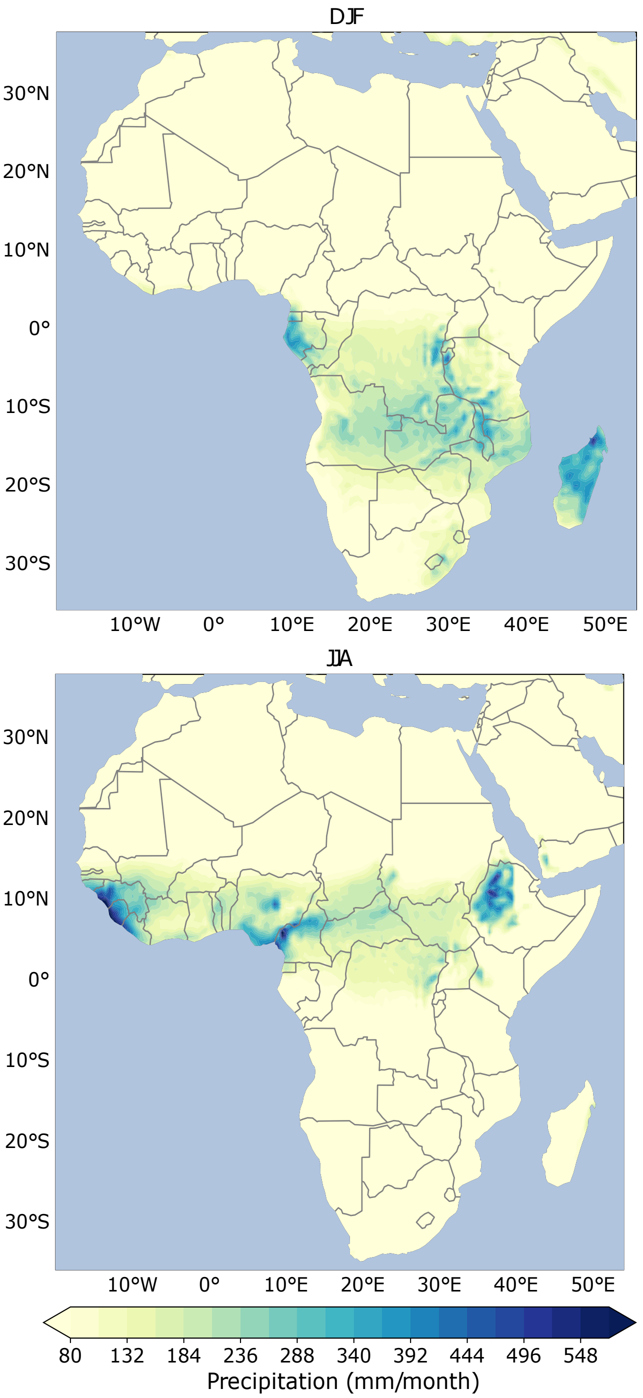

Figure S1. Mean ERA5 precipitation for the period 1979-2007 (mm/month) for Dec-Jan-Feb (top left), Mar-Apr-May (top right), Jun-Jul-Aug (bottom left), Sep-Oct-Nov (bottom right). Figure generated with python 3.8.6 [https://www.python.org/downloads/release/python-386/].

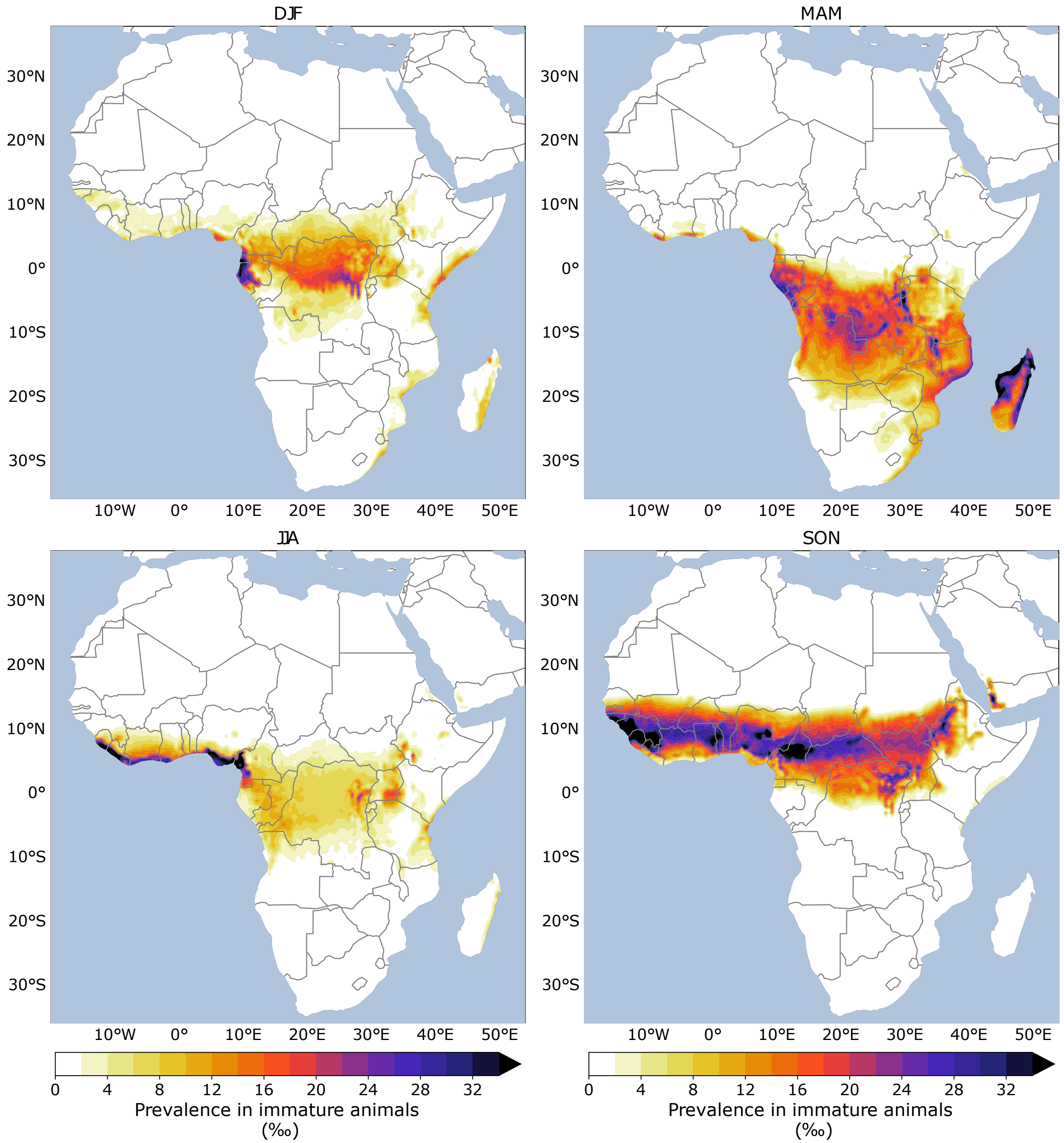

Figure S2. Mean simulated prevalence in immature animals based on ERA5 data for the period 1979-2007 (per thousand) for Dec-Jan-Feb (top left), Mar-Apr-May (top right), Jun-Jul-Aug (bottom left), Sep-Oct-Nov (bottom right). Figure generated with python 3.8.6 [https://www.python.org/downloads/release/python-386/].

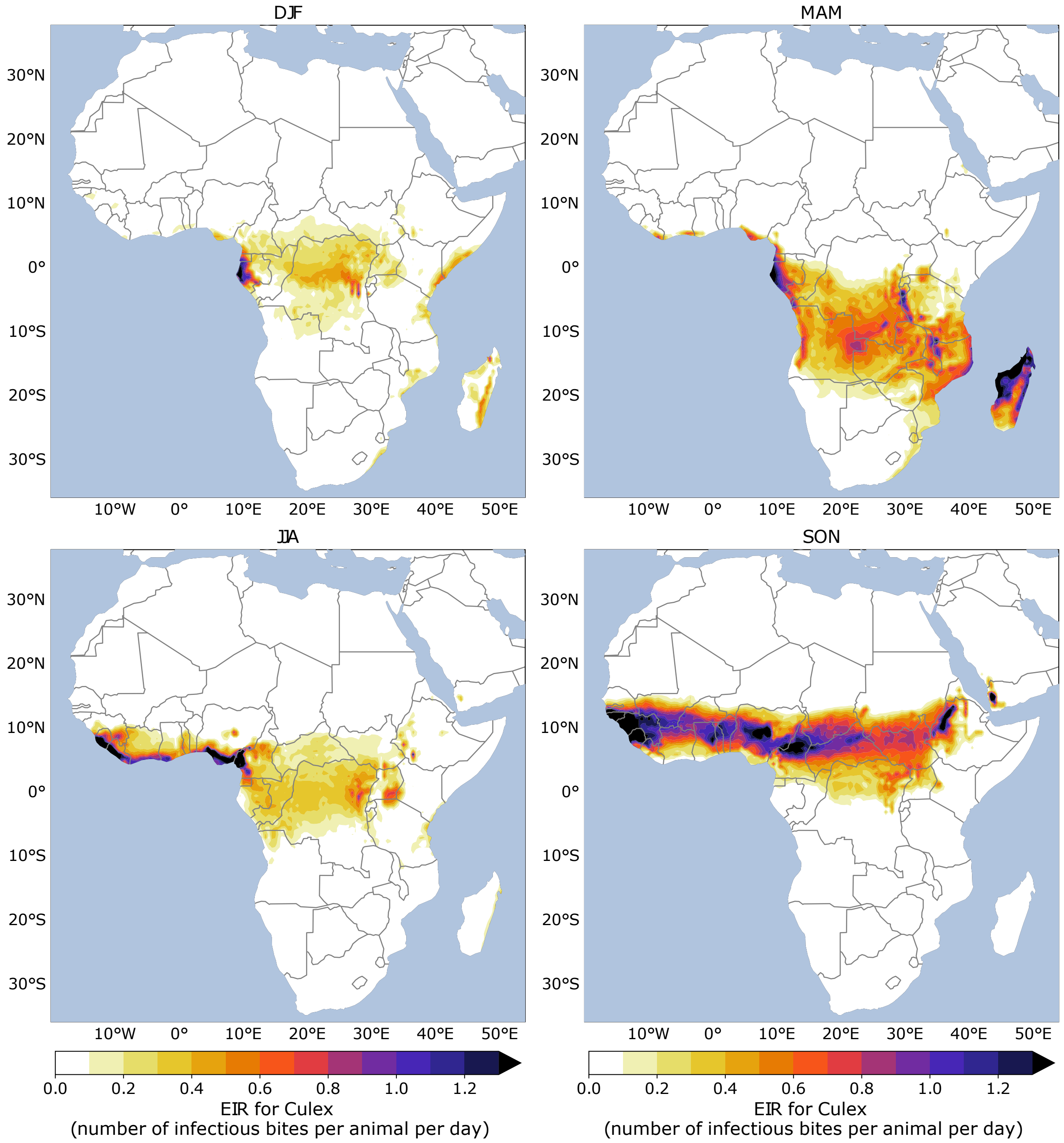

Figure S3. Mean simulated EIR for *Culex* based on ERA5 data for the period 1979-2007 (per thousand) for Dec-Jan-Feb (top left), Mar-Apr-May (top right), Jun-Jul-Aug (bottom left), Sep-Oct-Nov (bottom right). Figure generated with python 3.8.6 [https://www.python.org/downloads/release/python-386/].

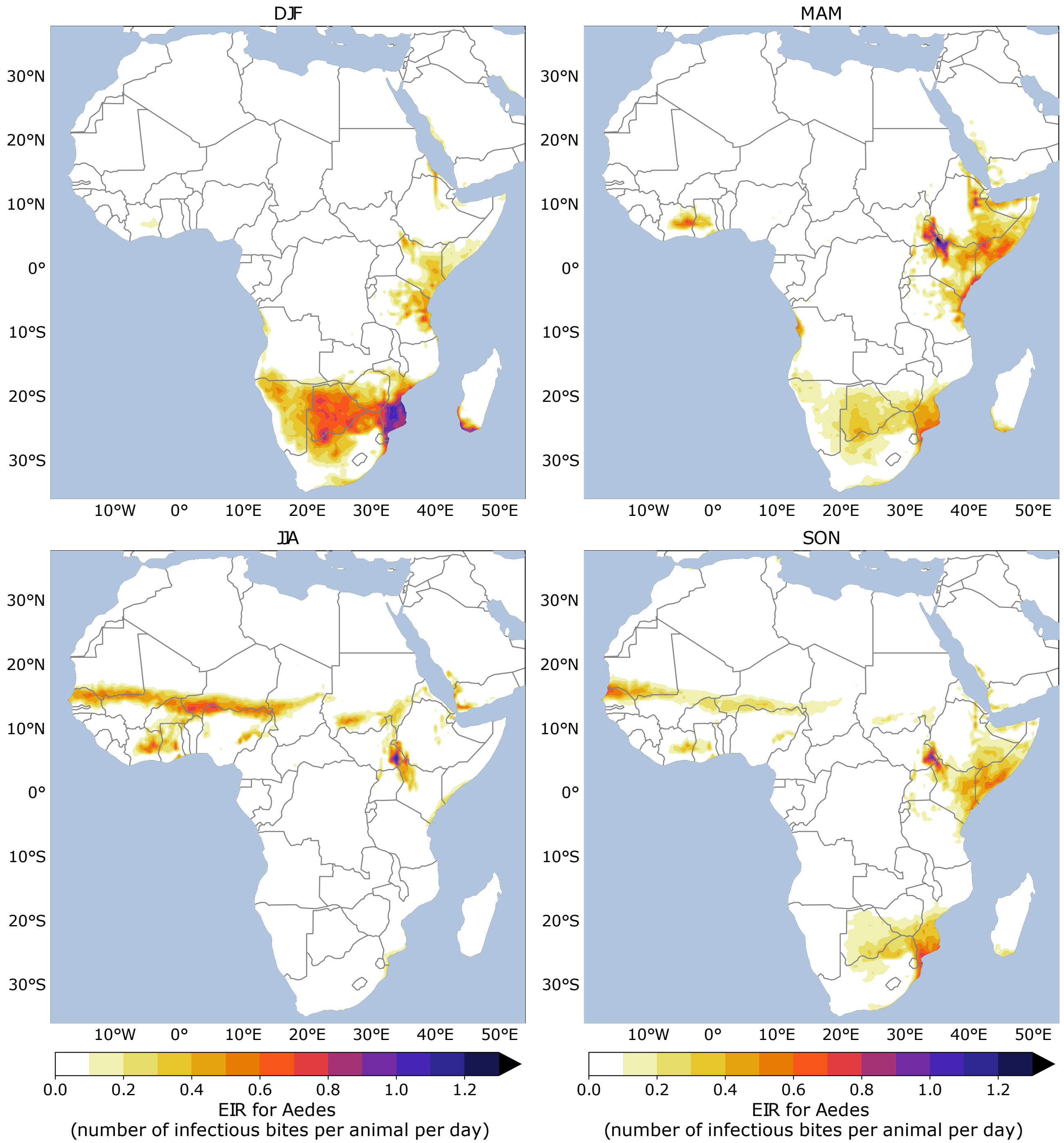

Figure S4. Mean simulated EIR for *Aedes* based on ERA5 data for the period 1979-2007 (per thousand) for Dec-Jan-Feb (top left), Mar-Apr-May (top right), Jun-Jul-Aug (bottom left), Sep-Oct-Nov (bottom right). Figure generated with python 3.8.6 [https://www.python.org/downloads/release/python-386/].

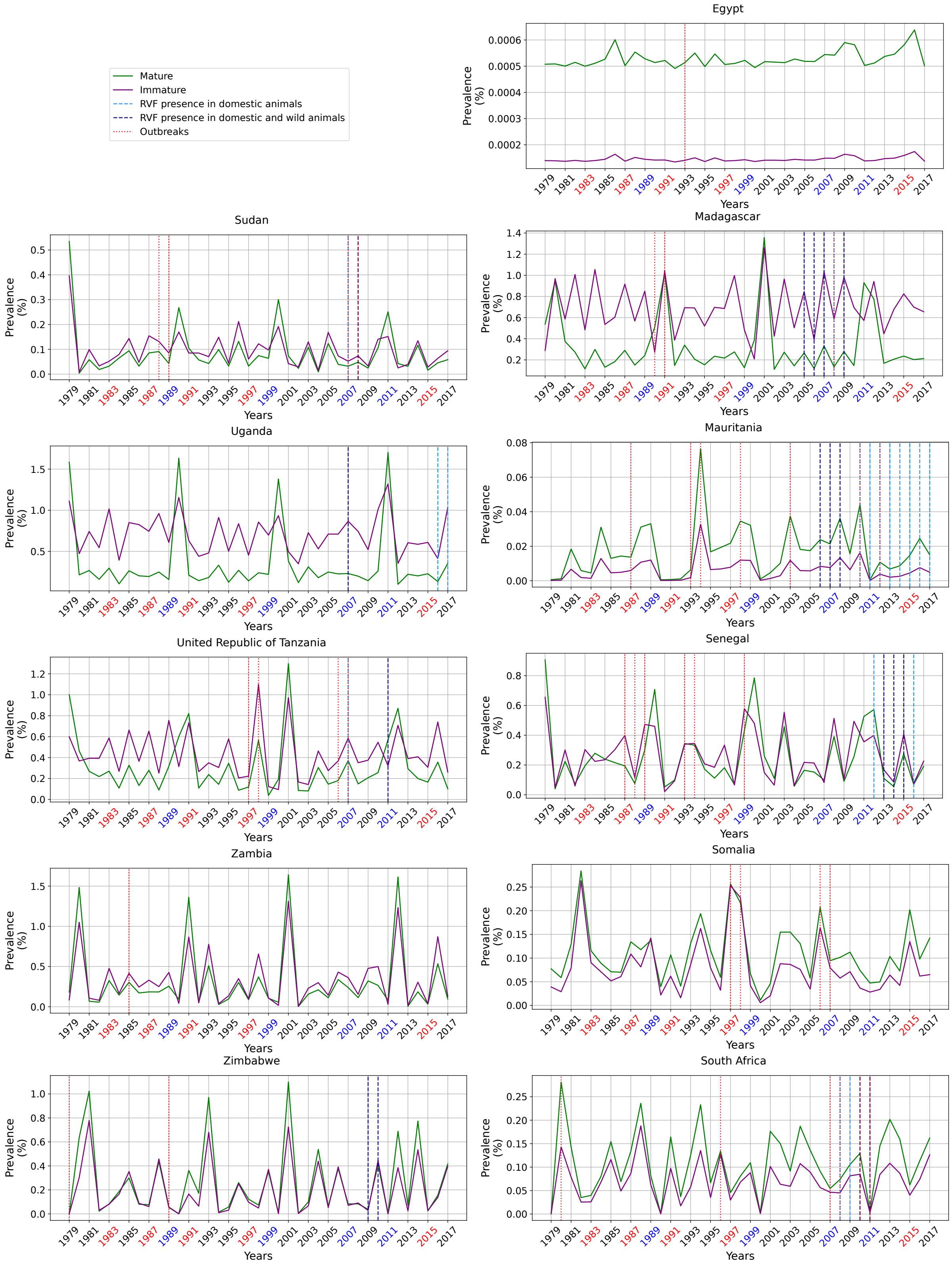

Figure S5. Simulated prevalence based on ERA5 data in mature (green solid line) and immature (purple solid line) livestock in African countries for 1979-2017 superimposed on observed outbreaks from Nanyingi et al.,[1] (red dotted line) for the period 1979-2014. Presence of the virus detected in domestic animals (WOAH data) is shown by the light blue dashed line and in domestic and wild animals is depicted by the dark blue dashed line for the period 2005-2018; see methods for further details. Figure generated with python 3.8.6 [https://www.python.org/downloads/release/python-386/].

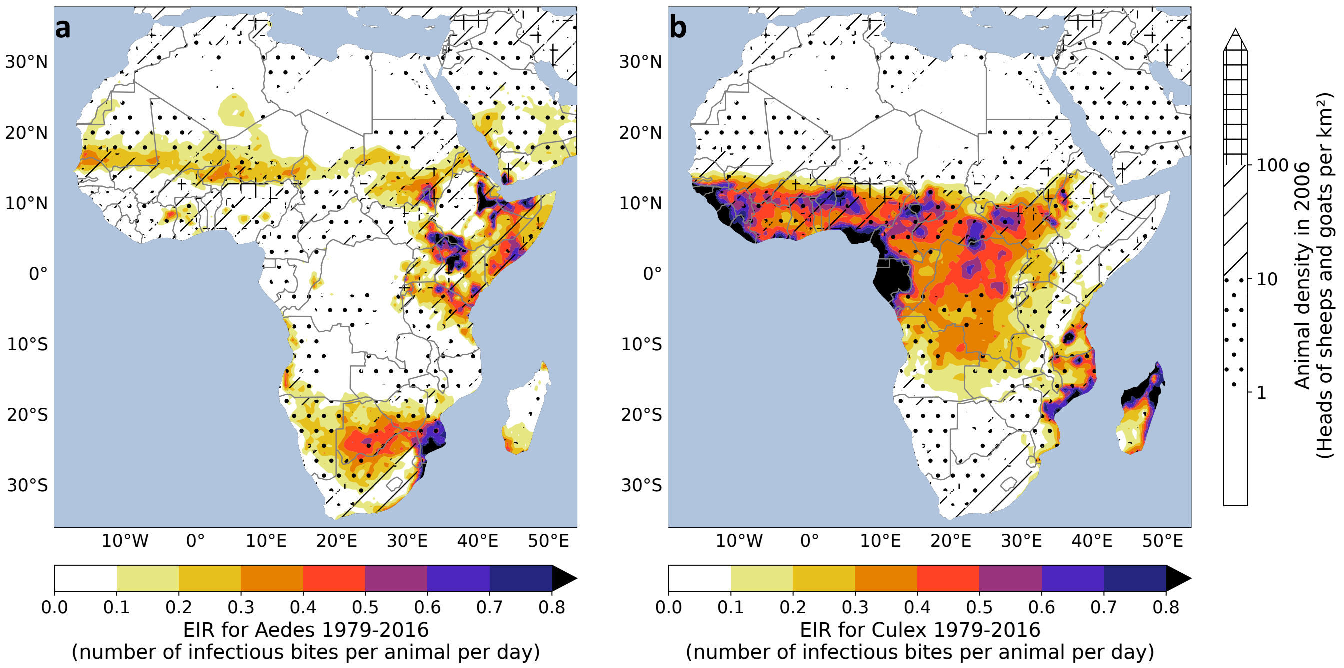

Figure S6. Simulated EIR based on EWEMBI data for a) *Aedes* and b) *Culex* in number of infectious bites per animal per day, averaged over the period 1979-2017 (color shading) superimposed on the number of goats and sheep per km^2 for the year 2006. The dots correspond to animal density ranging between 1 and 10 animals per km^2, the crosshatch to a density ranging between 10 and 100 animals per km^2 and the grid to a density above 100 animals per km^2. Figure generated with python 3.8.6 [https://www.python.org/downloads/release/python-386/].

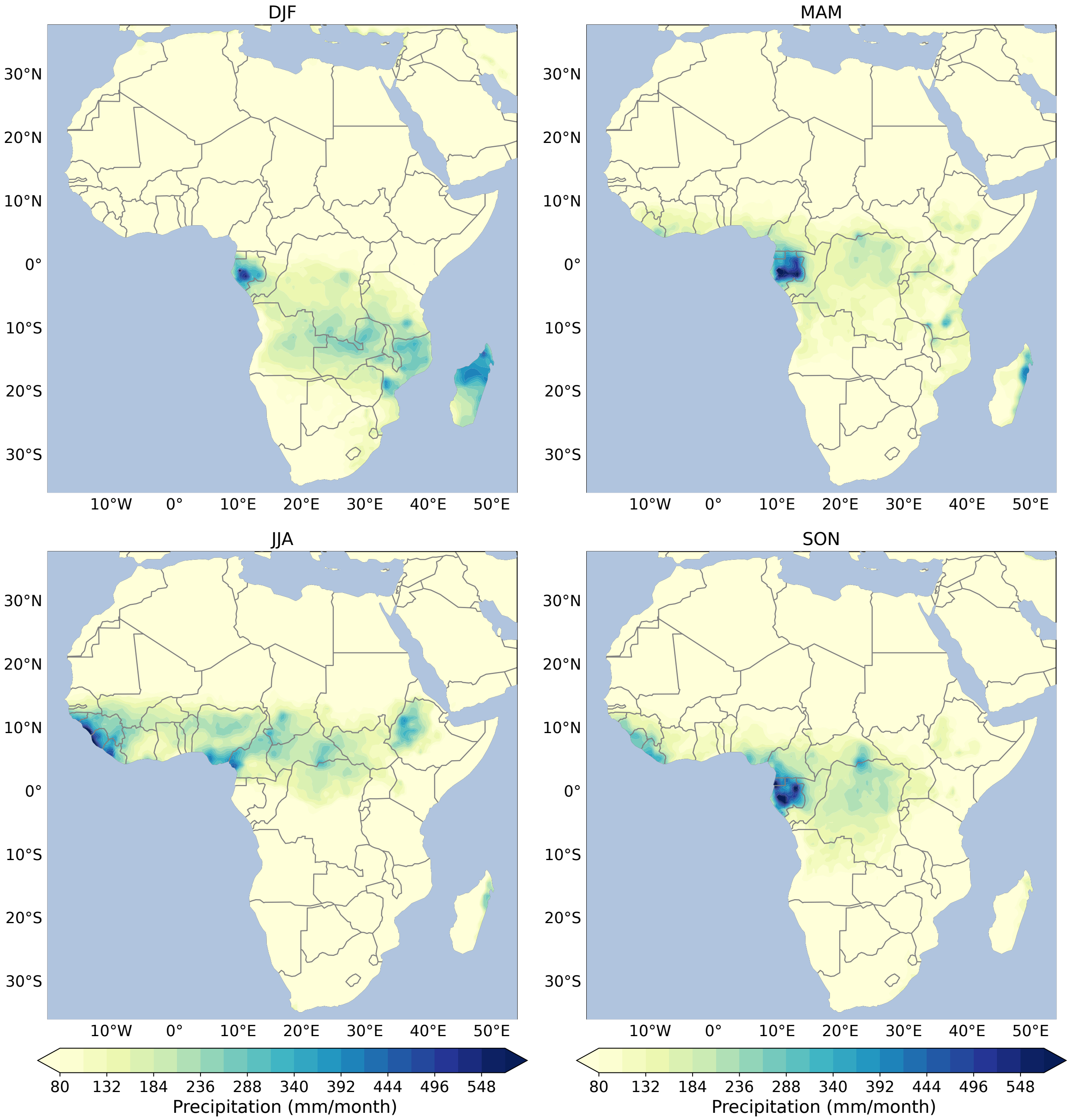

Figure S7. Mean EWEMBI rainfall for the period 1979-2007 (mm/month) for Dec-Jan-Feb (top left), Mar-Apr-May (top right), Jun-Jul-Aug (bottom left), Sep-Oct-Nov (bottom right). Figure generated with python 3.8.6 [https://www.python.org/downloads/release/python-386/].

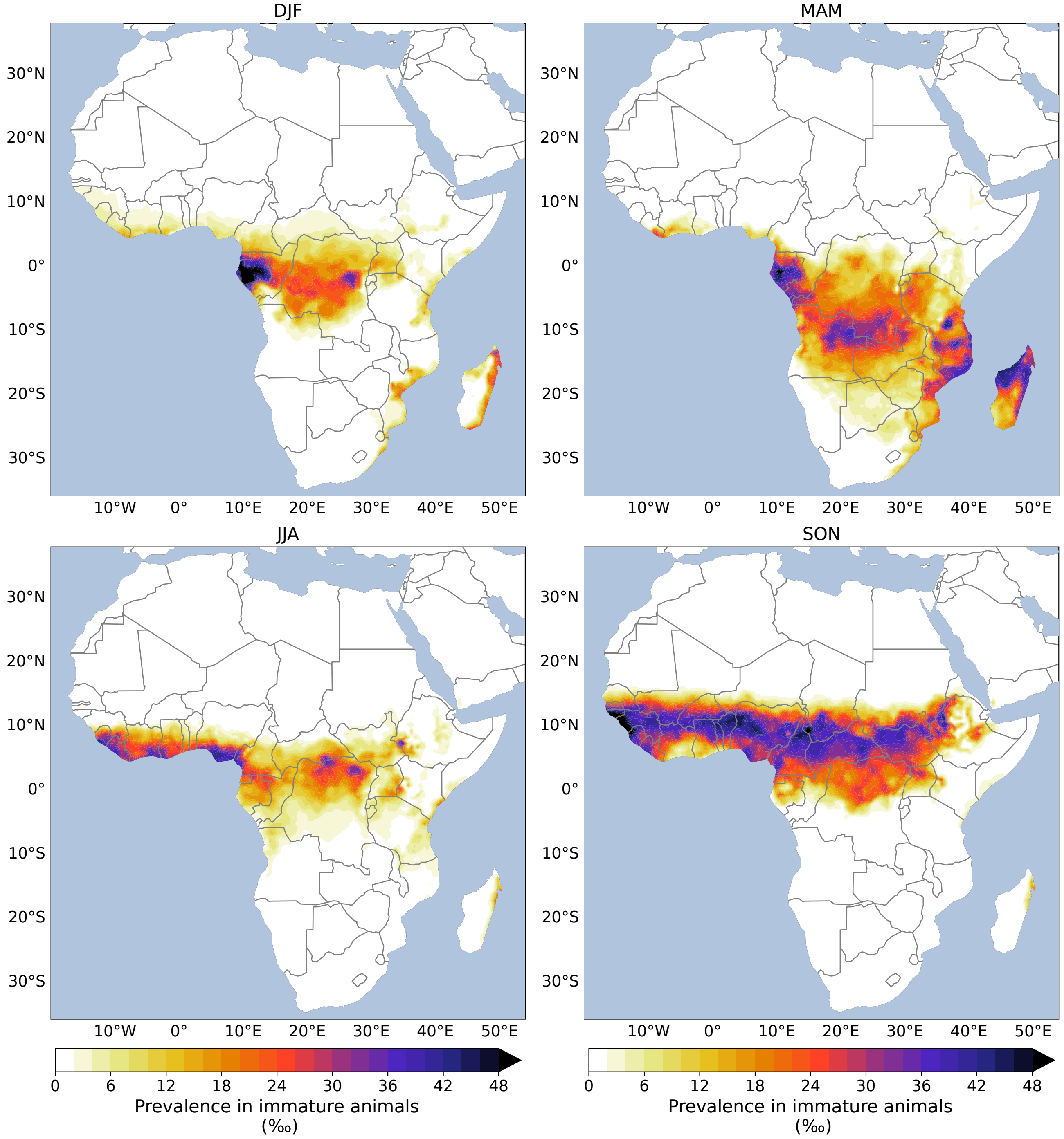

Figure S8. Mean simulated prevalence in immature animals based on EWEMBI data for the period 1979-2007 (per thousand) for Dec-Jan-Feb (top left), Mar-Apr-May (top right), Jun-Jul-Aug (bottom left), Sep-Oct-Nov (bottom right). Figure generated with python 3.8.6 [https://www.python.org/downloads/release/python-386/].

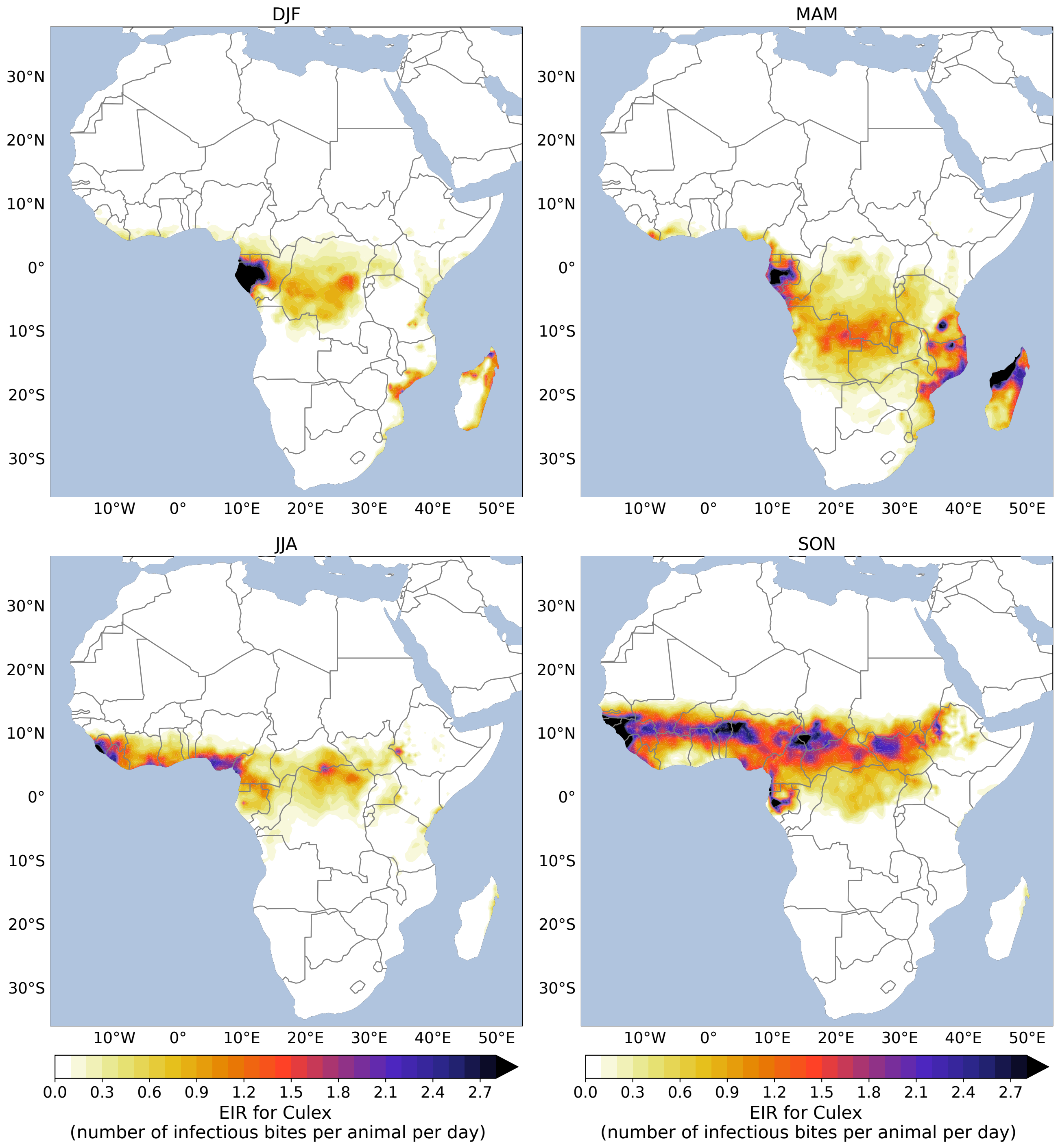

Figure S9. Mean simulated EIR for *Culex* based on EWEMBI data for the period 1979-2007 (per thousand) for Dec-Jan-Feb (top left), Mar-Apr-May (top right), Jun-Jul-Aug (bottom left), Sep-Oct-Nov (bottom right). Figure generated with python 3.8.6 [https://www.python.org/downloads/release/python-386/].

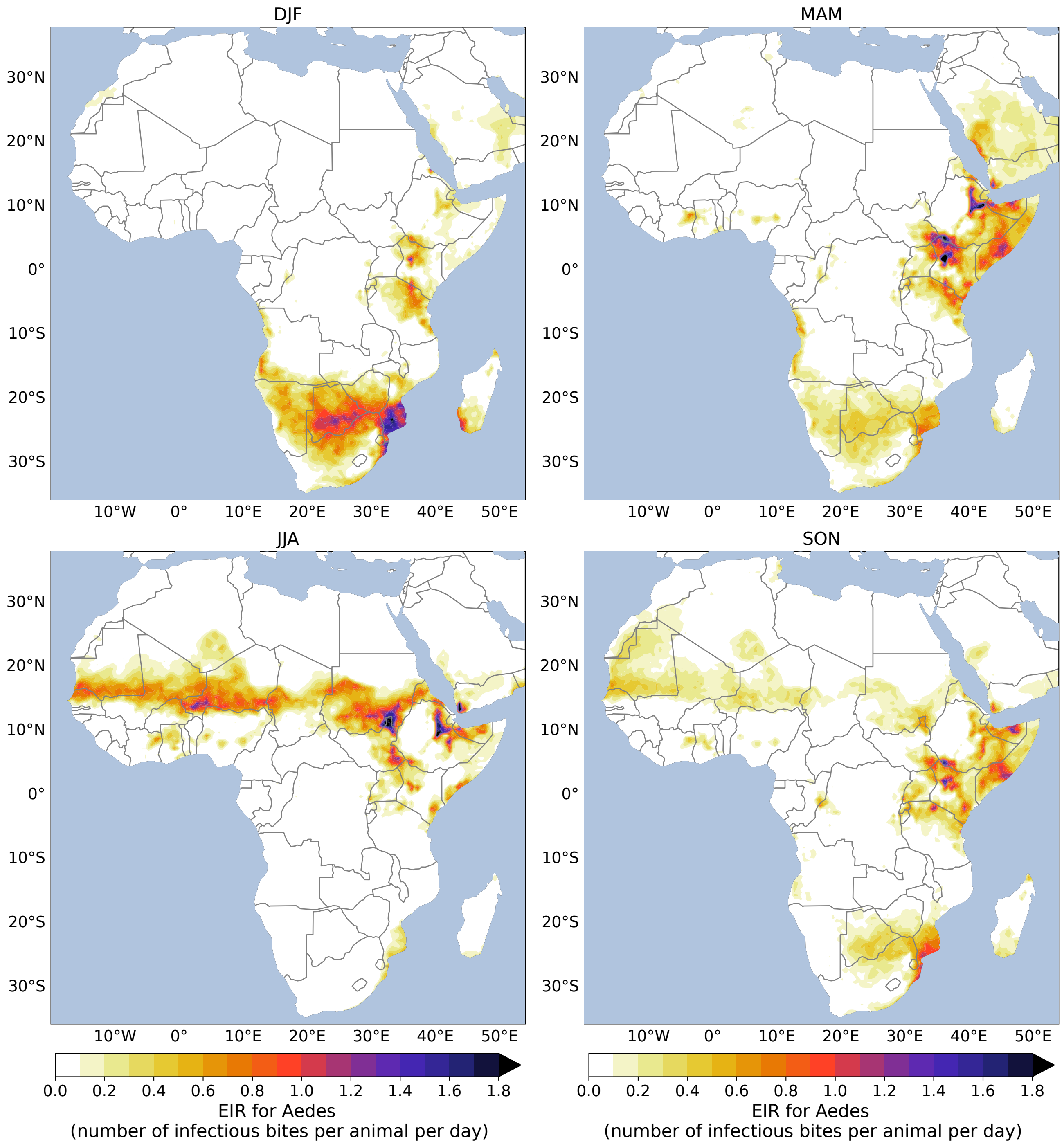

Figure S10. Mean simulated EIR for *Aedes* based on EWEMBI data for the period 1979-2007 (per thousand) for Dec-Jan-Feb (top left), Mar-Apr-May (top right), Jun-Jul-Aug (bottom left), Sep-Oct-Nov (bottom right). Figure generated with python 3.8.6 [https://www.python.org/downloads/release/python-386/].

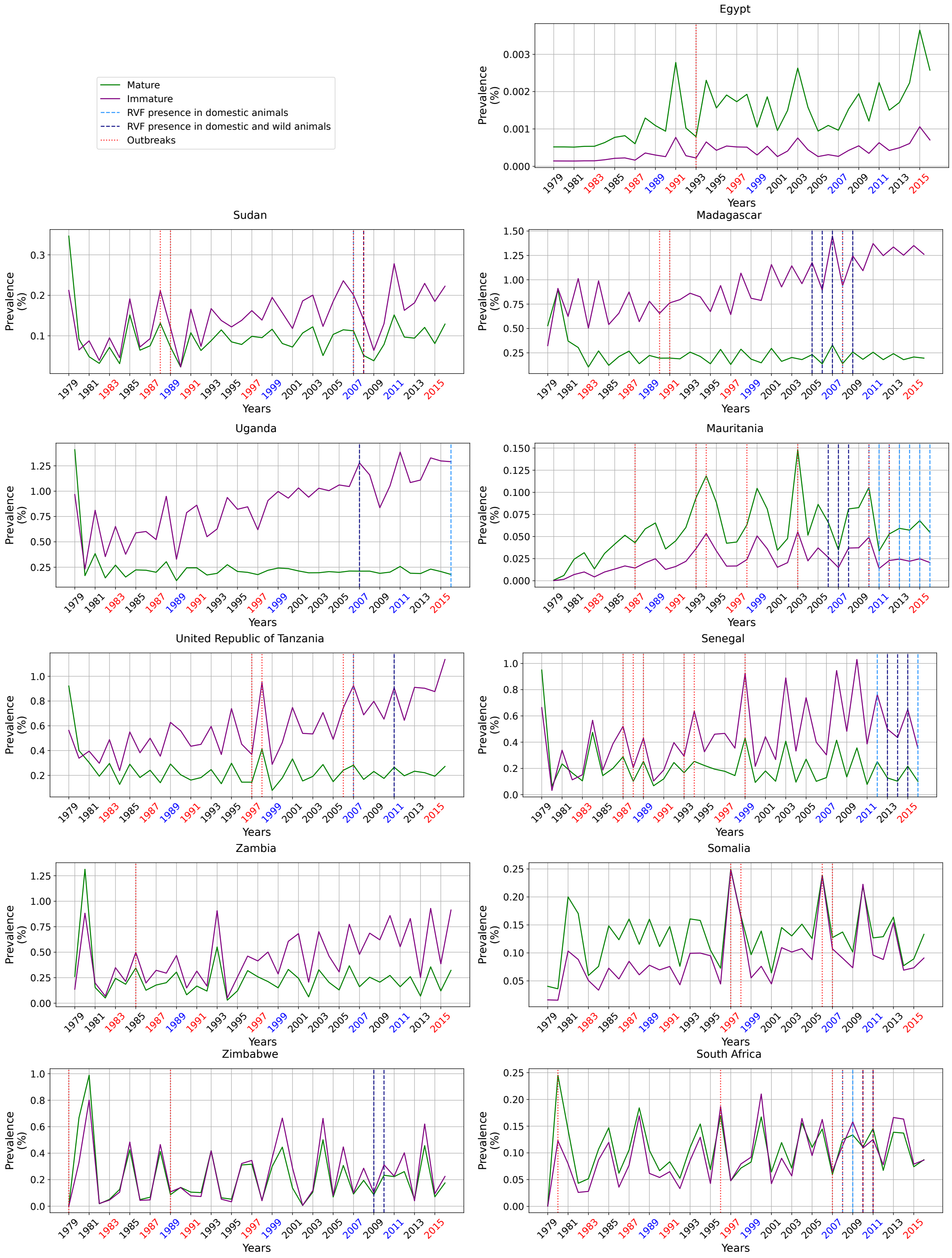

Figure S11. Simulated prevalence based on EWEMBI data in mature (green solid line) and immature (purple solid line) livestock in African countries for 1979-2017 superimposed on observed outbreaks from Nanyingi et al.,[1] (red dotted line) for the period 1979-2014. Presence of the virus detected in domestic animals (WOAH data) is shown by the blue dashed line for the period 2005-2018; see methods for further details. Figure generated with python 3.8.6 [https://www.python.org/downloads/release/python-386/].

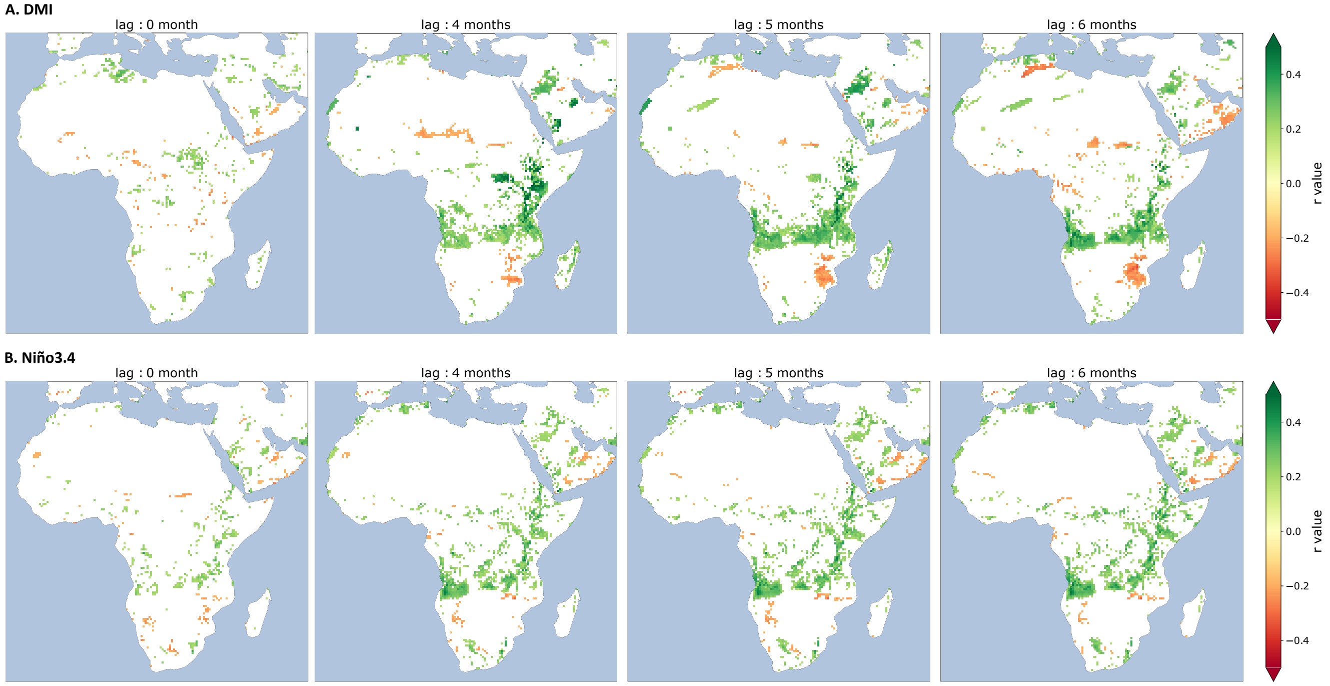

Figure S12. Lagged correlations between the DMI index (top), the Niño3.4 index (bottom) and simulated prevalence in immature livestock during the Mar-Avr-May season for the period 1979-2017. The SST indices have no lags (left panels), then the indices are leading simulated prevalence by four months (left middle panels), five months (right middle panels) and six months (right panels). Only correlation significant at the 95% confidence interval are shown. Figure generated with python 3.8.6 [https://www.python.org/downloads/release/python-386/].

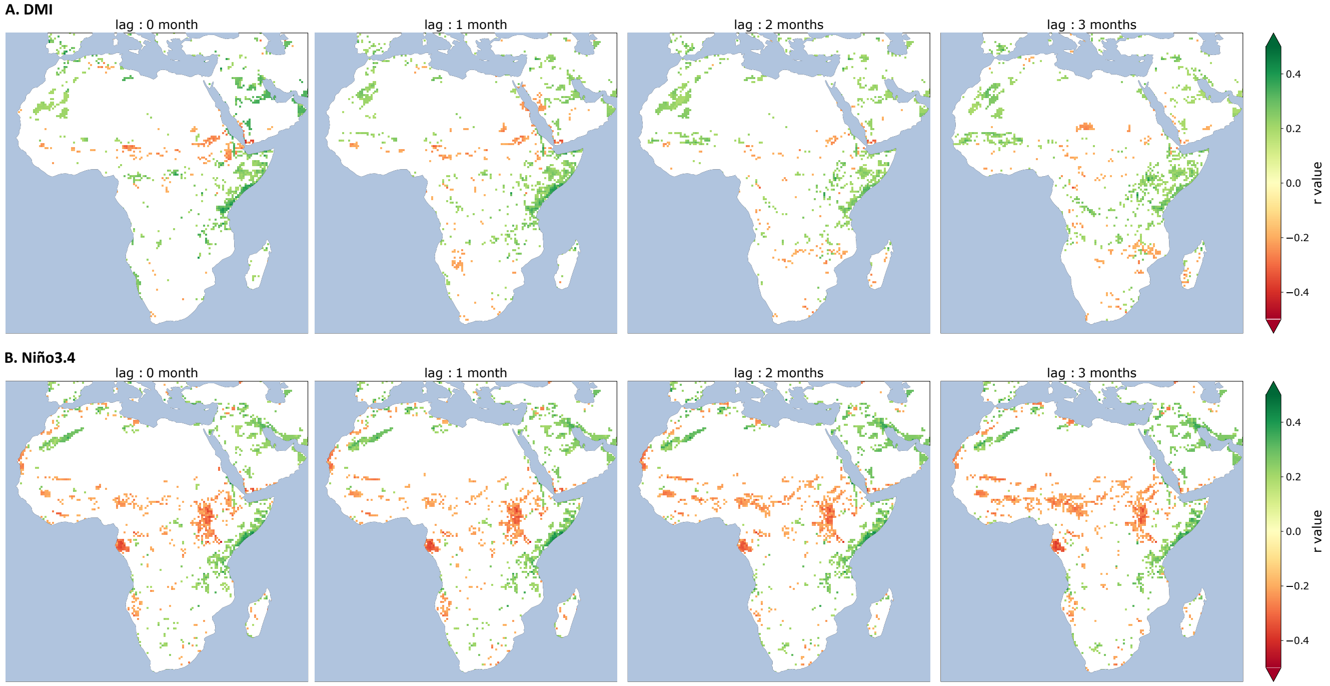

Figure S13. Lagged correlations between the DMI index (top), the Niño3.4 index (bottom) and simulated prevalence in immature livestock during the Sep-Oct-Nov season for the period 1979-2017. The SST indices have no lags (left panels), then the indices are leading simulated prevalence by one month (left middle panels), two months (right middle panels) and three months (right panels). Only correlation coefficients significant at the 95% confidence interval are shown. Figure generated with python 3.8.6 [https://www.python.org/downloads/release/python-386/].

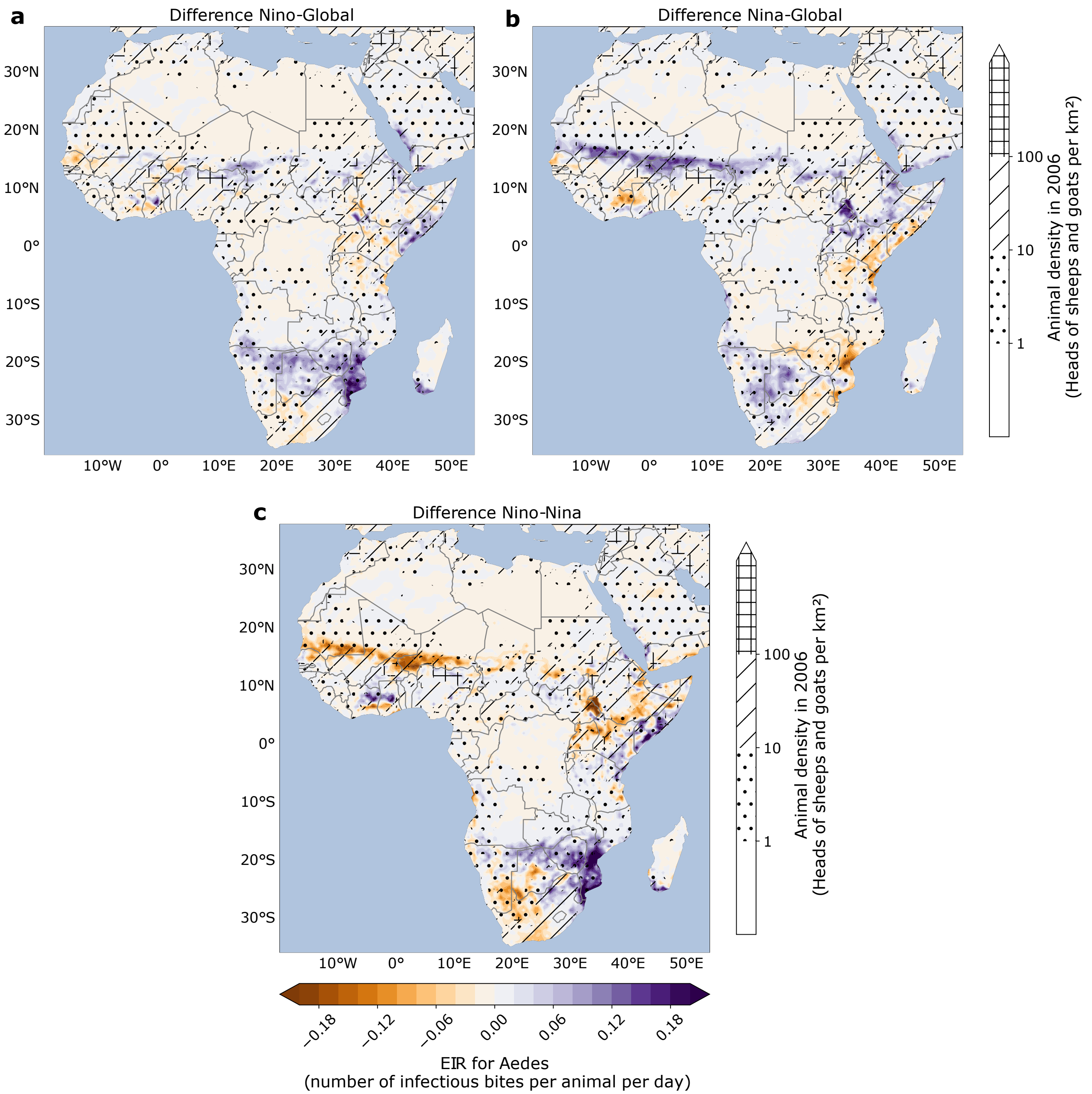

Figure S14. Composites of simulated EIR for *Aedes* (bites per animal per day) during a) El Niño events, b) La Niña events and c) difference in simulated EIR between El Niño and La Niña years. See Methods for details about the selection criteria of El Niño and La Niña years . Figure generated with python 3.8.6 [https://www.python.org/downloads/release/python-386/].

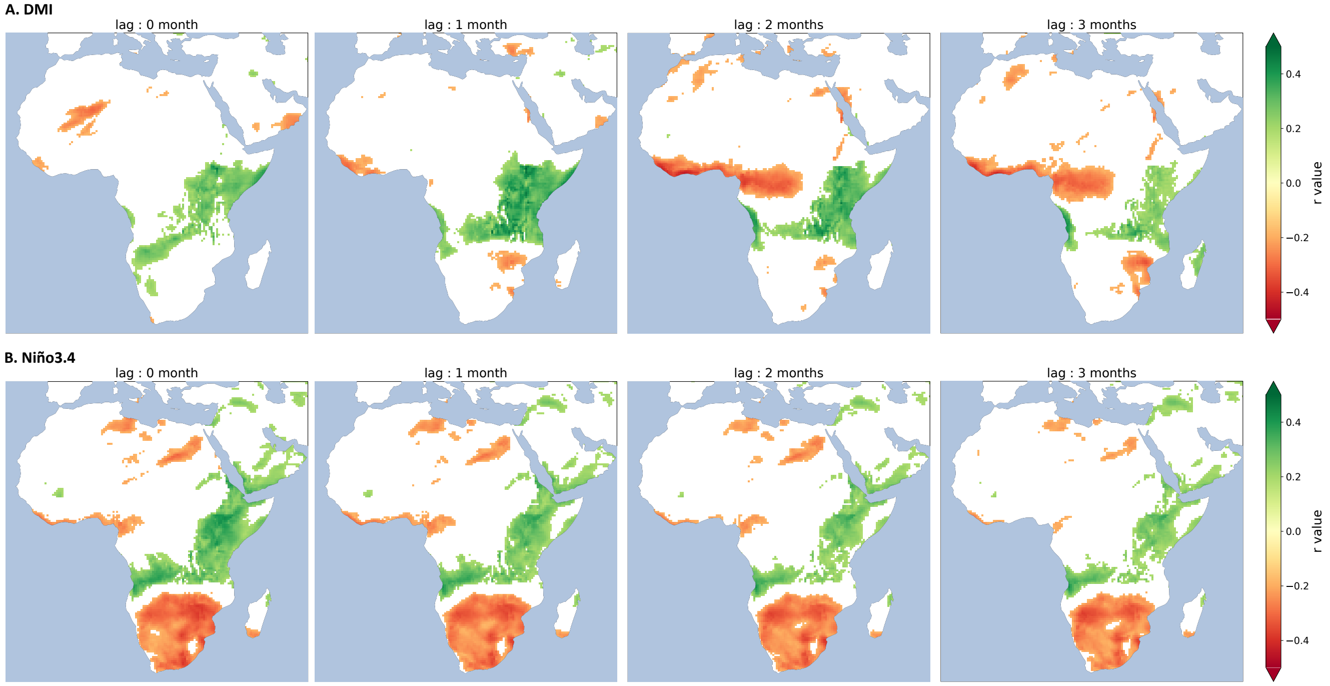

Figure S15. Lagged correlations between the DMI index (top), the Niño3.4 index (bottom) and precipitation from ERA5 data during the Dec-Jan-Feb season for the period 1979-2017. The SST indices have no lags (left panels), then the indices are leading precipitation by one month (left middle panels), two months (right middle panels) and three months (right panels). Only correlations significant at the 95% confidence interval are shown. Figure generated with python 3.8.6 [https://www.python.org/downloads/release/python-386/].

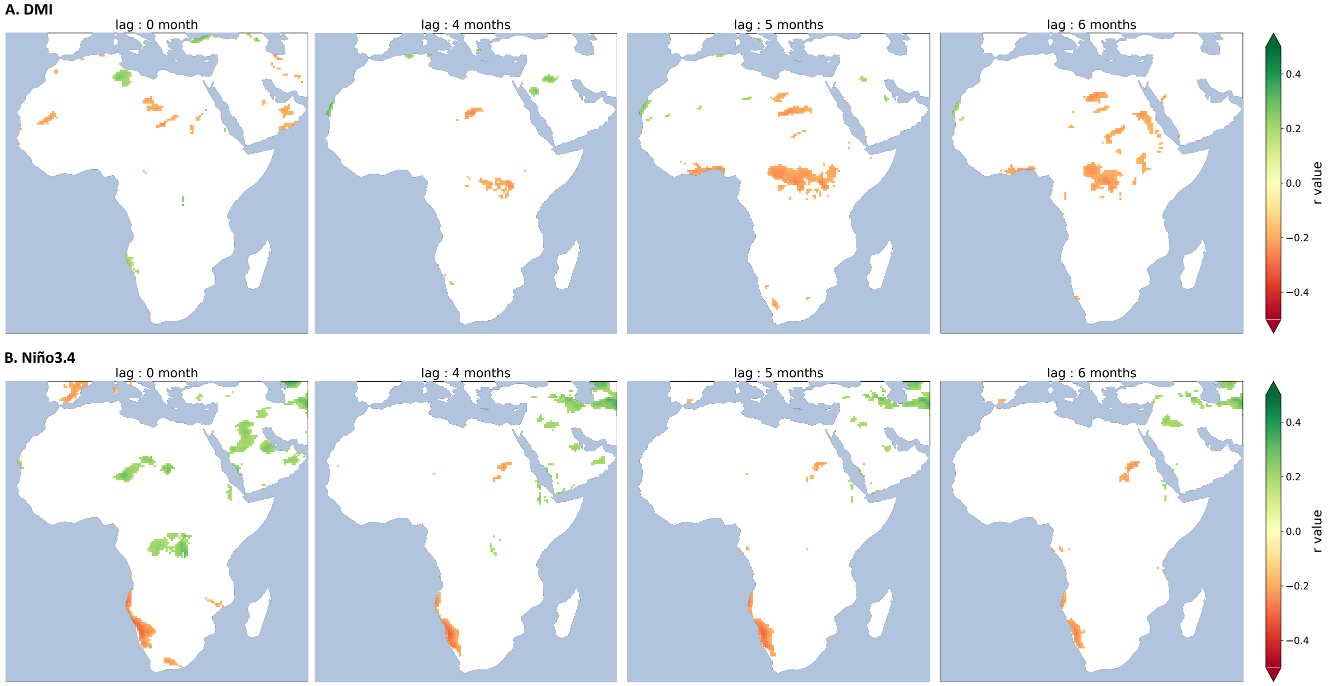

Figure S16. Lagged correlations between the DMI index (top), the Niño3.4 index (bottom) and precipitation from ERA5 data during the Mar-Avr-May season for the period 1979-2017. The indices have no lags (left panels), then the indices are leading precipitation by four months (left middle panels), five months (right middle panels) and six months (right panels). Only correlation significant at the 95% confidence interval are shown. Figure generated with python 3.8.6 [https://www.python.org/downloads/release/python-386/].

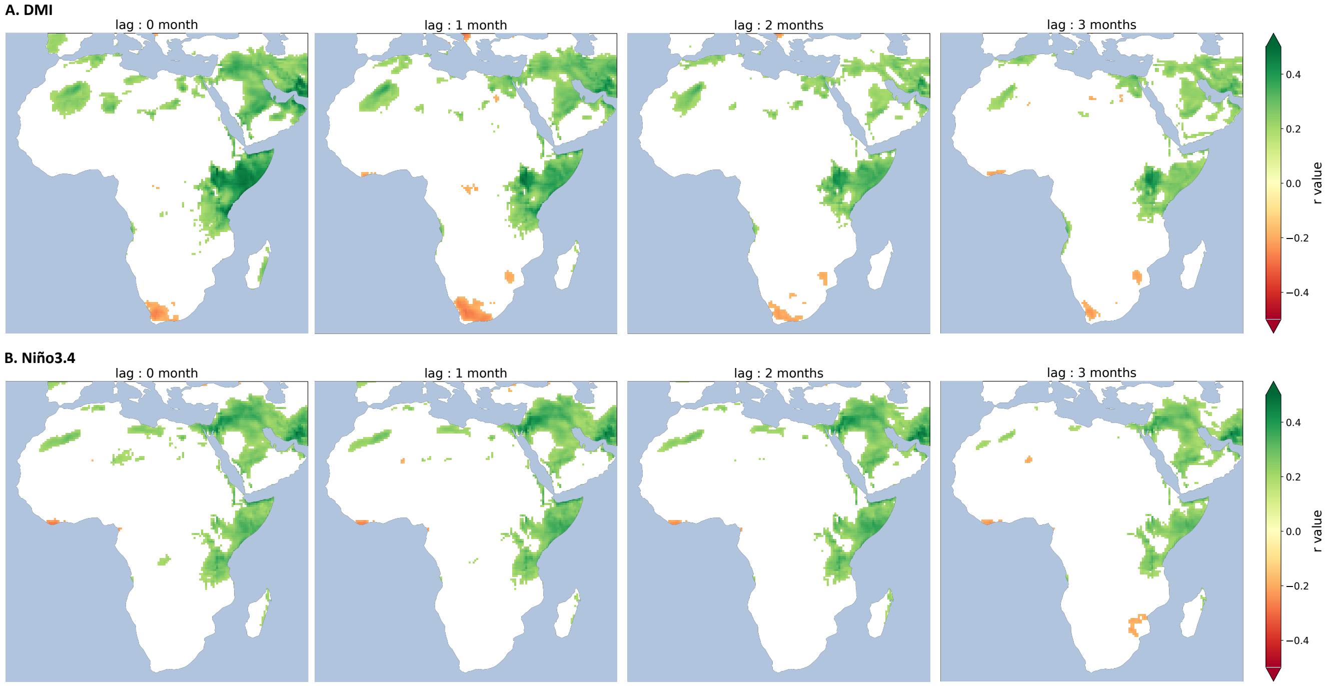

Figure S17. Lagged correlations between the DMI index (top), the Niño3.4 index (bottom) and ERA5 precipitation during the Sep-Oct-Nov season for the period 1979-2017. The indices have no lags (left panels), then the indices are leading precipitation by one month (left middle panels), two months (right middle panels) and three months (right panels). Only correlations significant at the 95% confidence interval are shown. Figure generated with python 3.8.6 [https://www.python.org/downloads/release/python-386/].

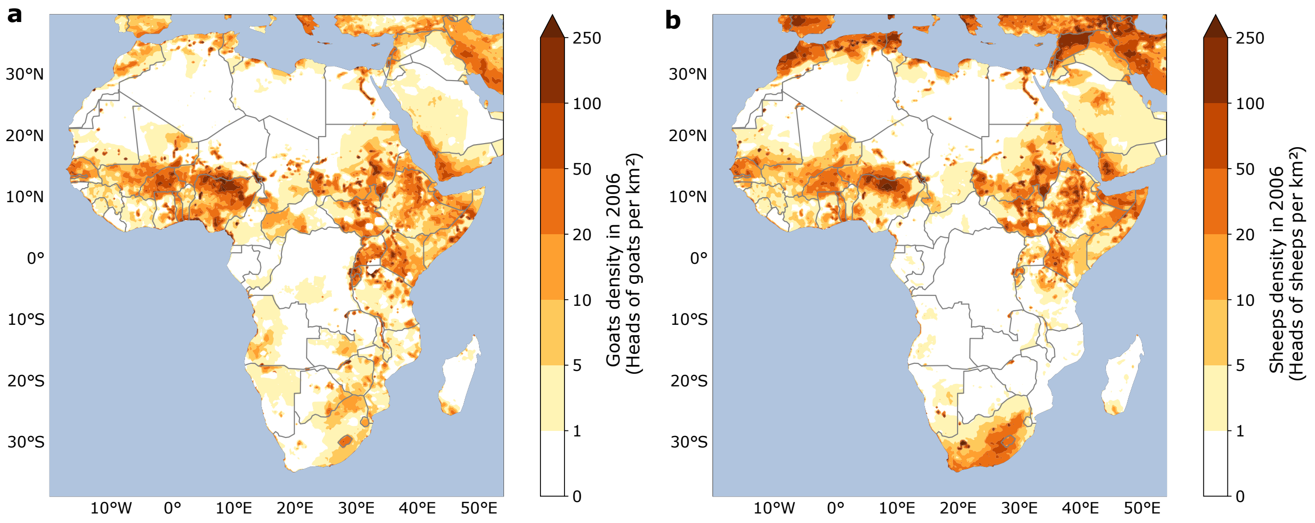

Figure S18. Density of a) goats and b) sheep for 2006 (number per km^2). Figure generated with python 3.8.6 [https://www.python.org/downloads/release/python-386/].

References

1. Nanyingi, M. O. *et al.* A systematic review of rift valley fever epidemiology 1931–2014. *Infect. ecology & epidemiology* **5**, 28024 (2015).
